# TNFα and IL-17 alkalinize airway surface liquid through CFTR and pendrin

**DOI:** 10.1152/ajpcell.00112.2020

**Published:** 2020-05-20

**Authors:** Tayyab Rehman, Ian M. Thornell, Alejandro A. Pezzulo, Andrew L. Thurman, Guillermo S. Romano Ibarra, Philip H. Karp, Ping Tan, Michael E. Duffey, Michael J. Welsh

**Affiliations:** ^1^Department of Internal Medicine, Roy J. and Lucille A. Carver College of Medicine, University of Iowa, Iowa City, Iowa; ^2^Howard Hughes Medical Institute, Roy J. and Lucille A. Carver College of Medicine, University of Iowa, Iowa City, Iowa; ^3^Department of Physiology and Biophysics, Jacobs School of Medicine and Biomedical Sciences, University at Buffalo, Buffalo, New York; ^4^Department of Molecular Physiology and Biophysics, Roy J. and Lucille A. Carver College of Medicine, University of Iowa, Iowa City, Iowa

**Keywords:** airway epithelia, anion secretion, inflammatory cytokines, pH, SLC26A4

## Abstract

The pH of airway surface liquid (ASL) is a key factor that determines respiratory host defense; ASL acidification impairs and alkalinization enhances key defense mechanisms. Under healthy conditions, airway epithelia secrete base (${\text{HCO}}_{3}^{-}$) and acid (H^+^) to control ASL pH (pH_ASL_). Neutrophil-predominant inflammation is a hallmark of several airway diseases, and TNFα and IL-17 are key drivers. However, how these cytokines perturb pH_ASL_ regulation is uncertain. In primary cultures of differentiated human airway epithelia, TNFα decreased and IL-17 did not change pH_ASL_. However, the combination (TNFα+IL-17) markedly increased pH_ASL_ by increasing ${\text{HCO}}_{3}^{-}$ secretion. TNFα+IL-17 increased expression and function of two apical ${\text{HCO}}_{3}^{-}$ transporters, CFTR anion channels and pendrin Cl^−^/${\text{HCO}}_{3}^{-}$ exchangers. Both were required for maximal alkalinization. TNFα+IL-17 induced pendrin expression primarily in secretory cells where it was coexpressed with CFTR. Interestingly, significant pendrin expression was not detected in CFTR-rich ionocytes. These results indicate that TNFα+IL-17 stimulate ${\text{HCO}}_{3}^{-}$ secretion via CFTR and pendrin to alkalinize ASL, which may represent an important defense mechanism in inflamed airways.

## INTRODUCTION

The pH of airway surface liquid (ASL), the thin layer of fluid that covers the airway epithelium, is a key factor that determines airway host defense (70, 85). Two of the main defense mechanisms in human airways are mucociliary clearance and antimicrobial factor-mediated bacterial killing. Previous studies have shown that abnormal acidification of the ASL impairs these processes, whereas acute alkalinization enhances them and may have therapeutic potential (2, 9, 19, 66, 77, 80).

Airway epithelia control the pH of ASL (pH_ASL_) through a balance between acid and base secretion (27). In proximal airways under normal, healthy conditions, acid secretion occurs primarily through H^+^/K^+^ ATPase (ATP12A) (20, 77), although other pathways may also be involved, including monocarboxylate transporters (MCT) (29), H^+^ channels (HVCN1), Na^+^/H^+^ exchangers (NHE3), and other H^+^-pumps (V-type ATPase) (27). Base (${\text{HCO}}_{3}^{-}$) secretion occurs primarily through CFTR anion channels (50, 69, 81), although Ca^2+^-activated anion channels (TMEM16A), the SLC26A9 anion transporter, and the Cl^−^/${\text{HCO}}_{3}^{-}$ countertransporter pendrin (SLC26A4) have also been reported to play a role to a varying extent (8, 13, 28, 41, 44, 49, 64).

In a wide variety of respiratory disorders, inflammatory conditions can also involve airway epithelia (61). In several airway diseases [cystic fibrosis (CF), certain endotypes of asthma, chronic obstructive pulmonary disease (COPD), and non-CF bronchiectasis], the inflammatory phenotype is neutrophil predominant (7, 12, 18, 21, 24, 51, 75). Cytokine and chemokine-driven neutrophil influx acts as a host-protective mechanism (5, 22, 60). However, how pH_ASL_ is regulated under such inflammatory conditions remains uncertain (15, 35, 43, 58, 65, 73, 84). For example, whether inflammation increases or decreases pH_ASL_ is not well established and may depend on species, models, and inflammatory stimuli (30, 33, 44). Additionally, the acid-base transport pathways modulated by inflammation are not precisely defined and vary from study to study. Moreover, how various epithelial cell types (ciliated cells, secretory cells, etc.) contribute to ${\text{HCO}}_{3}^{-}$ and H^+^ transport under inflammatory states is uncertain.

To better understand whether inflammation changes the way that airway epithelia control pHASL, we tested two key cytokines, tumor necrosis factor-α (TNFα) and interleukin-17 (IL-17). These cytokines have well-established roles in promoting neutrophilic inflammation (53, 56, 86). TNFα and IL-17 expression is increased in airways of people with CF, severe asthma, and COPD (25, 34, 57, 72, 87, 92). Moreover, their levels correlate with airway disease severity, rise with exacerbation, and fall with resolution of exacerbation (39, 57, 89). Additionally, approved agents that target TNFα and IL-17 pathways are clinically available, providing opportunities to test these agents for therapeutic intervention (32, 55). For these reasons, understanding how TNFα and IL-17 regulate pH_ASL_ is clinically relevant.

Here, we tested the hypothesis that TNFα and IL-17 regulate pH_ASL_. We used primary cultures of differentiated human airway epithelia grown at the air-liquid interface. We found that TNFα and IL-17 increased pH_ASL_ and investigated the cellular and molecular pathways involved. Learning how airway epithelia regulate pH_ASL_ in inflamed airways may lead to a better understanding of the pathogenesis of airway disorders and thereby guide future therapeutic strategies.

## MATERIALS AND METHODS

### 

#### Cell culture.

Primary cultures of differentiated airway epithelia were obtained without passage from multiple human donors (ages 15–73 yr; 49% male, 51% female) as previously reported (38). Briefly, donor tracheae and/or proximal bronchi were enzymatically digested and seeded together when both were available. Epithelial cells were isolated and seeded onto collagen-coated inserts (Costar no. 3470 polyester, no. 3460 polyester, no. 3413 polycarbonate). Epithelia were differentiated at the air-liquid interface for 3 wk or more before assay. All studies were approved by the University of Iowa Institutional Review Board.

To assess cytokine-induced responses, epithelia were treated on the basolateral side with 10 ng/mL TNFα (R&D Systems), 20 ng/mL IL-17 (R&D Systems), or both for 24 or 48 h based on dose-ranging and time-course studies. The physiologically relevant concentrations of TNFα and IL-17 would be those in the tissue compartment close to the epithelial basolateral membrane where receptors for these cytokines reside. That is also where studies have shown inflammatory cells expressing these cytokines in airway diseases (4, 25, 26, 34, 87). However, concentrations in that tissue compartment remain unknown. Although bronchoalveolar lavage (BAL) does not measure that space, studies of airway diseases have reported abnormally elevated concentrations of both TNFα and IL-17 in sputum and BAL liquid. For example, studies have reported IL-17 levels of 30–45 pg/mL in sputum (6, 57) and 10–15 pg/mL in BAL (87, 89) and TNFα levels of 90 pg/mL to 26–1,990 ng/L in sputum (39, 57). Given the tiny volume of ASL (~1 μL/cm^2^) and the large volume of BAL aliquots used in bronchial segment or subsegment, ASL dilution may well be >1,000-fold. Moreover, the concentrations within the epithelial compartment are likely to be higher. Of note, these concentrations of TNFα (10 ng/mL) and IL-17 (20 ng/mL) are similar to those reported by others who investigated their effects (16, 37, 44, 47, 57, 67). Subsequent studies revealed that these concentrations were adequate to investigate the mechanisms underlying pH_ASL_ alkalinization.

#### Pharmacologic reagents.

All drugs were purchased from Sigma-Aldrich except the following: GlyH-101 was a gift from the Cystic Fibrosis Foundation Therapeutics and Robert Bridges. VX-770 and VX-661 were purchased from Selleckchem and VX-445 from MedChemExpress.

#### pH_ASL_ measurement.

pH_ASL_ was assayed as previously reported (77). Briefly, we used a ratiometric pH indicator SNARF-1 conjugated to 70 kDa dextran (ThermoFisher Scientific). SNARF-1 is a single excitation (514 nm), dual emission (580 nm and 640 nm) fluorescence pH indicator with optimal range near physiologic pH. To minimize modification of ASL composition, SNARF-1, dextran was delivered as a powder to the apical side and allowed to distribute into ASL for 1 h. Fluorescence ratios were obtained on a laser scanning confocal microscope (Zeiss LSM 880) and converted to pH values using calibration curves constructed from colorless standard pH solutions. The microscope chamber housing epithelia maintained a humidified environment at 37°C. To mimic physiologic conditions, 5% CO_2_ was added to the chamber atmosphere whenever the basolateral side was immersed in an ${\text{HCO}}_{3}^{-}$ containing buffer solution but removed when an ${\text{HCO}}_{3}^{-}$-free buffer solution was used.

#### Ussing chamber studies.

Airway epithelia were mounted in modified Ussing chambers (Physiologic Instruments), and bathed in Krebs-Ringer buffer solution containing (in mM): 118.9 NaCl, 25 NaHCO_3_, 2.4 K_2_HPO_4_, 0.6 KH_2_PO_4_, 1.2 MgCl_2_, 1.2 CaCl_2_, 5 dextrose, at 37°C and adjusted to pH 7.4 in the presence of 5% CO_2_. Mucosal and serosal chambers were voltage clamped, followed by recording of short-circuit current (*I*_SC_) and transepithelial conductance (*G*_t_) at baseline and in response to the sequential apical addition of (in μM): 100 amiloride, 50 uridine triphosphate (UTP), 100 4,4'-diisothiocyano-2,2'-stilbenedisulfonic acid (DIDS), 10 forskolin and 100 3-isobutyl-2-methylxanthine (IBMX), and 100 GlyH-101.

#### Real-time PCR.

Total RNA was isolated from airway epithelia using RNeasy Lipid Tissue Mini Kit (QIAGEN). Genomic DNA was removed through DNase I (QIAGEN) treatment. Quality of RNA isolation was verified on NanoDrop 2000 spectrophotometer (ThermoFisher Scientific), and samples with 260/280 ratio ≥ 1.8 were carried forward. RNA was reverse transcribed with SuperScript VILO MasterMix (Invitrogen). cDNA thus obtained was amplified using gene-specific primers (Table 1) and Fast SYBR Green Master Mix (Applied Biosystems) on QuantStudio 6 Flex Real-Time PCR System (Applied Biosystems). All reactions were performed as triplicate and gene expression was quantitated as fold change (2^−ΔΔCT^).

**Table 1.** Primers used for real-time PCR studies

Gene	Primer	Reference
*SCNN1A*	GAACAACTCCAACCTCTGGATGTC TCTTGGTGCAGTCGCCATAAT	11
*TMEM16A*	GCCATGAACTCCTCCCC ACAAACCGGCCTTTGAAGAA	77
*CFTR*	CACCCAGCCATTTTTGGC AGGAGCGATCCACACGAA	77
*SLC26A4*	CTCCCCAAATACCGAGTCAA CCATATCCGACAGGAACTGC	91
*β-Actin*	AGAGCTACGAGCTGCCTGAC AGCACTGTGTTGGCGTACAG	93

#### siRNA knockdown.

Gene knockdown in primary airway epithelia was achieved as reported previously (71). Negative control and gene-specific siRNAs were obtained from Integrated DNA Technologies (Table 2), and transfected into dissociated primary airway epithelial cells using Lipofectamine RNAiMax (Invitrogen). Epithelia were seeded onto collagen-coated inserts (Costar #3470), and differentiated at the air-liquid interface. pH_ASL_ was measured at day 6 or 7 past seeding. The efficiency of gene knockdown was assessed with real-time PCR.

**Table 2.** siRNAs used for knockdown studies

Target	Duplex Sequence
*CFTR*	(IDT# hs.Ri.CFTR.13.2) 5′-rGrUrCrArUrCrArArArGrCrArUrGrCrCrArArCrUrArGrAAG-3′ 5′-rCrUrUrCrUrArGrUrUrGrGrCrArUrGrCrUrUrUrGrArUrGrArCrGrC-3′
*SLC26A4*	(IDT# hs.Ri.SLC26A4.13.2) 5′-rArCrUrCrUrCrArUrUrCrArGrGrArUrUrGrUrArArArGrATA-3′ 5′-rUrArUrCrUrUrUrArCrArArUrCrCrUrGrArArUrGrArGrArGrUrGrA-3′
Negative Control	(IDT# DS NC 1)

#### RNA-sequencing protocol and analysis.

RNA-sequencing (RNA-seq) was performed by the University of Iowa Genomics Division using manufacturer-recommended protocols. Briefly, 500 ng of DNase I-treated total RNA was enriched for polyA containing transcripts using beads coated with oligo(dT) primers. The enriched RNA pool was fragmented, converted to cDNA, and ligated to sequencing adaptors using the Illumina TruSeq stranded mRNA sample preparation kit (Illumina no. RS-122-2101). The molar concentrations of the indexed libraries were measured using the 2100 Bioanalyzer (Agilent) and combined equally into pools for sequencing. The concentrations of the pools were measured with the Illumina Library Quantification Kit (KAPA Biosystems) and sequenced on the Illumina HiSeq 4000 genome sequencer using 75 bp paired-end SBS chemistry.

Pseudoalignment of raw sequencing reads and quantification of transcript-level expression were obtained using Kallisto version 0.45.0 and human transcriptome reference GRCh38.p12 (10). Gene counts were imported into R, and differential expression tests were performed using DESeq2 version 1.22.2 (52). Furthermore, gene expression modeling in DESeq2 accounted for the experimental design, acknowledging and correcting for paired control and treated samples for each donor.

To assess cytokine-induced changes in ${\text{HCO}}_{3}^{-}$ transport, a gene ontology-based approach was used. Gene ontology accession for bicarbonate transport was identified with The Gene Ontology Resource (http://geneontology.org/). The accession term (GO:0015701) was used to mine BioMart (http://useast.ensembl.org/biomart/martview/6e73b1adcd14c5c772bc0ead86726df6) and obtain a data set of bicarbonate transport-related genes. This data set was further expanded and refined through a literature search. The results were visualized as a heatmap generated with Clustvis tool (https://biit.cs.ut.ee/clustvis/) (59).

#### Intracellular pH assay.

Airway epithelia were washed thrice with the Krebs-Ringer solution and loaded with 5 μM BCECF-AM (ThermoFisher Scientific) in the presence of 2.5 mM probenecid to prevent dye extrusion. After 40 min of incubation, epithelia were washed again and immediately transferred to a custom-made chamber on the Zeiss LSM 880 confocal microscope. The basolateral side was submerged in the Krebs-Ringer solution. Using a perfusion pump, the apical side was superfused either with the Krebs-Ringer solution or a Cl^−^-free buffer solution containing (in mM): 118.9 Na gluconate, 25 NaHCO_3_, 2.4 K_2_HPO_4_, 0.6 KH_2_PO_4_, 1.0 Mg gluconate, and 5 Ca gluconate. The microscope chamber maintained a humidified, 5% CO_2_ atmosphere at 37°C. The final pH for all buffer solutions was 7.4. Imaging was performed with a ×40 water immersion lens, and BCECF fluorescence was continuously recorded as the apical buffer was switched from the Krebs-Ringer to Cl^−^-free buffer and finally back to the Krebs-Ringer.

BCECF is a dual excitation (440 nm, 490 nm), single emission (535 nm), ratiometric, pH-sensitive dye. To obtain intracellular pH (pH_i_) values from fluorescence emission ratios, standard curves were constructed. Briefly, after being loaded with BCECF-AM, epithelia were transferred to a high K^+^ calibration buffer containing (in mM): 120 KCl, 15 NaCl, 2.4 K_2_HPO_4_, 0.6 KH_2_PO_4_, 1.2 MgCl_2_, 1.2 CaCl_2_, and 20 HEPES. The final pH was adjusted by adding HCl or KOH to cover a range from 6 to 8.5. To clamp pH_i_ to the same value as the basolateral buffer, epithelia were kept in known pH buffer solution in the presence of 10 μM nigericin for 10 min, and BCECF fluorescence was measured as described above. Standard curves were generated by plotting fluorescence emission ratios against known pH values.

#### Immunocytochemistry.

Airway epithelia were washed thrice with PBS, fixed with 4% paraformaldehyde for 15 min, and permeabilized with 0.3% Triton X-100 for 20 min. To minimize nonspecific staining, epithelia were treated with SuperBlock (ThermoFisher Scientific) containing 0.5% normal goat serum for 1 h at room temperature. Primary antibodies were diluted in SuperBlock and added apically for 3 h at 37°C. Epithelia were washed and incubated for 45 min with appropriate secondary antibodies diluted in PBS. The primary antibodies used included: mouse anti-SLC26A4 (1:200; Abnova cat. no. H00005172-A01), mouse anti-CFTR (1:100; R&D Systems cat. no. MAB25031), rabbit anti-acetyl-α-tubulin (1:500; Cell Signaling Technology cat. no. 5335), rat anti-SCGB1A1 (1:100; R&D Systems cat. no. MAB4218), and rabbit anti-BSND (1:100; Abcam cat. no. ab196017). For CFTR and pendrin colabeling studies, rabbit anti-SLC26A4 (1:200; Novus Biologicals cat. no. NBP1-60106) was used. To detect primary antibodies, the following secondary antibodies were used: goat anti-mouse, goat anti-rabbit or goat anti-rat conjugated to Alexa Flour 488 or 568 (1:1,000; ThermoFisher Scientific cat. no. A-11017, A-21069, A-11077). Actin cytoskeleton was stained with Alexa Fluor 633 phalloidin (1:300; ThermoFisher Scientific cat. no. A22284) added at the same time as secondary antibodies. Epithelia were mounted on glass slides, and Vectashield with DAPI (Vector Laboratories) was used to secure glass coverslips. Imaging was performed on the Olympus Fluoview FV 3000 confocal microscope. Z-stack images were processed with the Olympus Fluoview program.

#### Statistics.

Testing for statistical significance was performed on GraphPad Prism 8 (GraphPad Software). Tests included unpaired or paired *t* test for comparing two groups, repeated measures ANOVA with posttest Tukey’s or Dunnett’s correction for more than two groups, and Anderson–Darling test for normal distribution. A *P* value of < 0.05 was considered significant. Analysis of RNA-seq data is described above.

## RESULTS

### 

#### Combination of TNFα and IL-17 markedly increases pH_ASL_.

**Fig. 1.**
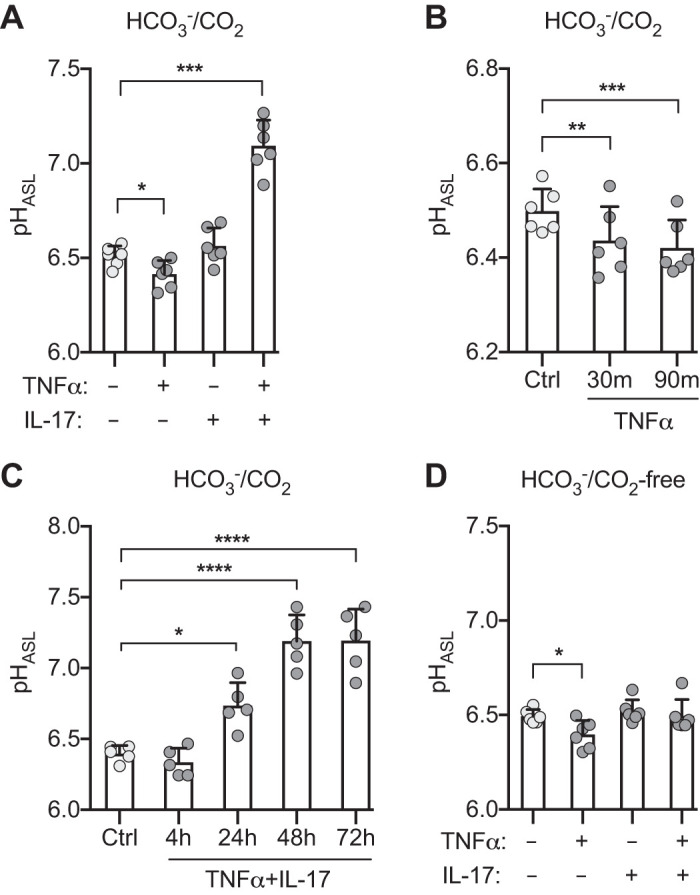
Combination of TNFα and IL-17 increases pH of airway surface liquid (pH_ASL_). Primary cultures of differentiated human airway epithelia were treated with TNFα (10 ng/mL), IL-17 (20 ng/mL), or both for the indicated times, and pH_ASL_ was measured using SNARF-1-dextran. *A*: ${\text{HCO}}_{3}^{-}$/CO_2_-containing Krebs-Ringer (*n* = 6). *B*: TNFα alone in ${\text{HCO}}_{3}^{-}$/CO_2_-containing Krebs-Ringer for indicated times (*n* = 6). *C*: TNFα and IL-17 in ${\text{HCO}}_{3}^{-}$/CO_2_-containing Ringers for indicated times (*n* = 5). *D*: ${\text{HCO}}_{3}^{-}$/CO_2_-free Krebs HEPES buffer (*n* = 6). Each data point represents epithelium from a different donor. Light and dark gray circles represent control and cytokine-treated epithelia, respectively. Bars indicate means and SD. *P < 0.05, **P < 0.01, ***P < 0.001, ****P < 0.0001 by repeated measures ANOVA and Tukey’s multiple comparisons test.

We treated primary cultures of differentiated human airway epithelia with two key proinflammatory cytokines, TNFα (10 ng/mL) and IL-17 (20 ng/mL). Twenty-four hours later, we measured pH_ASL_ in the presence of 25 mM ${\text{HCO}}_{3}^{-}$ and 5% CO_2_ (Fig. 1*A*). TNFα alone decreased pH_ASL_, whereas IL-17 alone produced no change. Because TNFα and IL-17 are both likely to be elevated in inflammatory airway disorders (7, 45, 56, 57), we also tested the combination. In striking contrast to the individual cytokines, the combination of TNFα+IL-17 markedly increased pH_ASL_. These results suggest that TNFα regulates pH_ASL_ differently than IL-17 and that TNFα and IL-17 signaling pathways interact to produce an unexpectedly large increase in pH_ASL_.

In subsequent studies, we also explored the time course of effects. Thirty minutes after TNFα was added, pH_ASL_ decreased and did not further change at 90 min. (Fig. 1*B*). This time course suggests regulation of H^+^ secretion through a posttranslational mechanism. In contrast, TNFα+IL-17 increased pH_ASL_ over a much slower time course (Fig. 1*C*), suggesting regulation by transcriptional mechanisms. In subsequent experiments, we applied 10 ng/mL TNFα and 20 ng/mL IL-17 and studied epithelia 24 h later to assess the initial response to cytokines and minimize secondary changes.

Because these studies were performed in an ${\text{HCO}}_{3}^{-}$/CO_2_ environment, pH_ASL_ could have increased due to increased ${\text{HCO}}_{3}^{-}$ secretion, decreased H^+^ secretion, or both. To begin to identify the underlying transport processes, we replaced basolateral ${\text{HCO}}_{3}^{-}$ with HEPES and removed CO_2_ from the atmosphere. As we observed in the presence of ${\text{HCO}}_{3}^{-}$/CO_2_, TNFα alone decreased pH_ASL_, indicating that TNFα acidifies ASL by increasing H^+^ secretion (Fig. 1*D*). IL-17 alone did not change pH_ASL_, which suggested that it did not alter H^+^ transport. Importantly, and in contrast to the large increase observed in the presence of ${\text{HCO}}_{3}^{-}$/CO_2_, TNFα+IL-17 failed to increase pH_ASL_. This result suggested that TNFα+IL-17 increased pH_ASL_ by increasing ${\text{HCO}}_{3}^{-}$ secretion and not by reducing H^+^ secretion.

#### TNFα+IL-17 increase CFTR-mediated HCO_3_^−^ secretion and ASL alkalinization.

**Fig. 2.**
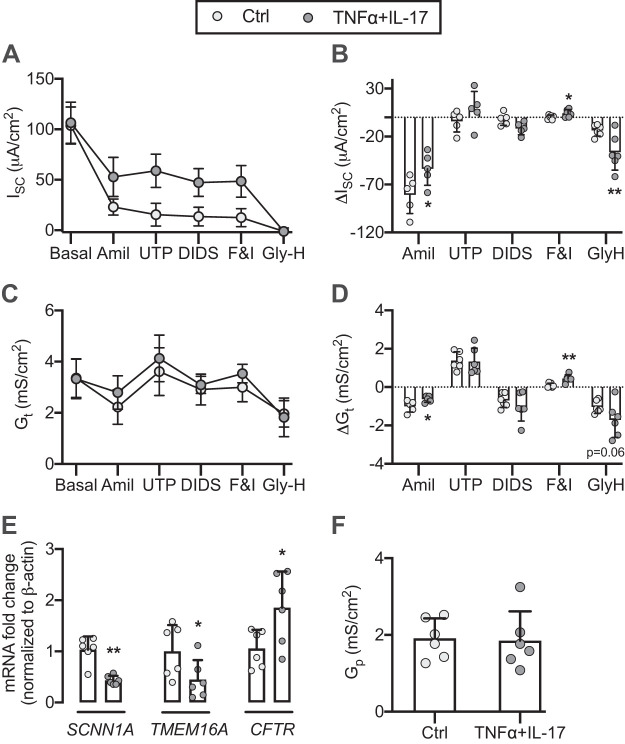
TNFα+IL-17 increase CFTR activity and expression. After TNFα+IL-17 treatment for 24 h, human airway epithelia were mounted in modified Ussing chambers with symmetric Krebs-Ringer solution gassed with 5% CO_2_. Epithelia were voltage clamped followed by continuous recording of short-circuit current (*I*_SC_) and transepithelial conductance (*G*_t_) as pharmacologic agents were sequentially added to the apical chamber. *A*–*D*: *I*_SC_, Δ*I*_SC_, *G*_t_, and Δ*G*_t_ in control and TNFα+IL-17-treated epithelia. *E*: TNFα+IL-17-induced changes in *SCNN1A*, *TMEM16A*, and *CFTR* transcript abundance. *F*: estimate of paracellular conductance (*G*_p_) obtained from residual *G*_t_ after inhibition of epithelial Na^+^ channel (ENaC), calcium-activated anion channel (CaCC), and CFTR. For *A*–*D*, *n* = 5 different donors; for *E* and *F*, *n* = 6 different donors. Bars indicate means and SD. Statistical significance between control and TNFα+IL-17-treated epithelia was tested using paired Student’s *t* test. *P < 0.05, **P < 0.01.

${\text{HCO}}_{3}^{-}$ secretion into ASL requires an apical membrane transport mechanism. Under basal conditions, this role is performed in large part by CFTR (50, 69, 81). However, a calcium-activated anion channel (CaCC, TMEM16A) might also mediate ${\text{HCO}}_{3}^{-}$ secretion under inflamed conditions (13, 30, 36). We asked whether TNFα+IL-17 altered the activity of these anion channels. We mounted epithelia in modified Ussing chambers with symmetrical Krebs-Ringer solution and measured *I*_SC_ and *G*_t_ responses to channel activators and inhibitors. TNFα+IL-17 reduced amiloride-sensitive *I*_SC_ and *G*_t_, suggesting reduced epithelial Na^+^ channel (ENaC) activity (Fig. 2, *A*–*D*). Although not statistically significant, TNFα+IL-17 tended to increase the response to UTP (a CaCC activator) and DIDS (a CaCC inhibitor), suggesting that CaCC may warrant further investigation. Compared with vehicle control, epithelia treated with TNFα+IL-17 had a larger change in *I_SC_* and *G*_t_ in response to forskolin/IBMX, which led to CFTR phosphorylation (23, 79) and activity, and GlyH-101, which inhibits CFTR channel activity (63). These results suggested that TNFα+IL-17 reduced ENaC-mediated Na^+^ absorption and increased CFTR-mediated anion secretion. Consistent with the electrophysical changes, TNFα+IL-17 reduced mRNA for *SCNN1A* (ENaC α-subunit) and *TMEM16A* but nearly doubled *CFTR* expression (Fig. 2*E*).

In addition to transcellular mechanisms, the paracellular pathway provides a route for transepithelial ion transport. We asked whether TNFα+IL-17 changed paracellular conductance. Because *G*_t_ is the sum of transcellular conductance (*G*_c_) and paracellular conductance (*G*_p_) [*G*_t_ = *G*_c_ + *G*_p_], reducing *G*_c_ to ~0 provides a residual *G*_t_ that approximates *G*_p_. In airway epithelia, *G*_c_ is chiefly dependent on apical conductance to Na^+^ (ENaC) and Cl^−^ (CFTR and CaCC). Therefore, residual *G*_t_ after blocking ENaC, CaCC, and CFTR provides an estimate of *G*_p_. TNFα+IL-17 for 24 h did not alter estimated *G*_p_ (Fig. 2*F*). This result suggested that increased ${\text{HCO}}_{3}^{-}$ secretion was likely not mediated via the paracellular route, with the caveat that the paracellular permeability to ${\text{HCO}}_{3}^{-}$ was not directly measured and could have changed in the absence of a change in *G*_p_. However, the transepithelial voltage (−31 mV), the estimated apical [${\text{HCO}}_{3}^{-}$] (10–12 mM at pH 7–7.1), and the basolateral [${\text{HCO}}_{3}^{-}$] (24 mM) suggest that the electrochemical driving forces in TNFα+IL-17-treated epithelia would favor paracellular ${\text{HCO}}_{3}^{-}$ absorption, not ${\text{HCO}}_{3}^{-}$ secretion.

**Fig. 3.**
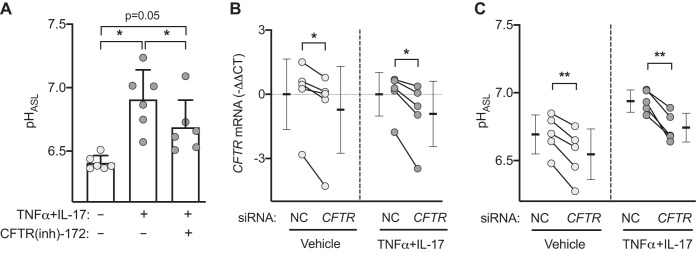
CFTR contributes to TNFα+IL-17-induced airway surface liquid (ASL) alkalinization. *A*: after TNFα+IL-17 treatment for 24 h, human airway epithelia were treated for 2 h with either apical vehicle (DMSO) or CFTR(inh)-172 suspended in a volatile solvent (Fluorinert FC-72) to achieve an approximate ASL concentration of 10 μM. pH of ASL (pH_ASL_) was measured with SNARF-1-dextran in the presence of ${\text{HCO}}_{3}^{-}$/CO_2_ (*n* = 6). *B* and *C*: effect of negative control (NC) or siRNA directed against *CFTR* on pH_ASL_ in vehicle- or TNFα+IL-17-treated epithelia (*n* = 5). Each data point represents epithelium from a different donor. Bars indicate means ± SD. Data were analyzed using repeated measures ANOVA with Tukey’s multiple comparisons test (*A*) or paired Student’s *t* test (*B* and *C*). *P < 0.05, **P < 0.01.

We asked whether CFTR contributed to the TNFα+IL-17-induced increase in pH_ASL_. After 24 h of TNFα+IL-17 treatment, we added either vehicle (DMSO) or CFTR inhibitor [CFTR(inh)-172] (suspended in a volatile solvent perfluorocarbon Fluorinert FC-72) to the ASL (Fig. 3*A*). CFTR inhibition significantly reduced the pH_ASL_ response but did not return it to control values without TNFα+IL-17. We also applied siRNA targeted to *CFTR*. *CFTR* knockdown reduced pH_ASL_ in both untreated and TNFα+IL-17-treated epithelia (Fig. 3, *B* and *C*). These results indicate that CFTR contributed to the TNFα+IL-17-induced increase in pH_ASL_. Because pH_ASL_ did not return to control levels after pharmacological or siRNA inhibition of CFTR, the data raised the possibility that TNFα+IL-17 might induce an additional ${\text{HCO}}_{3}^{-}$ secretion mechanism.

#### TNFα+IL-17 alkalinize ASL through CFTR and non-CFTR mechanisms.

**Fig. 4.**
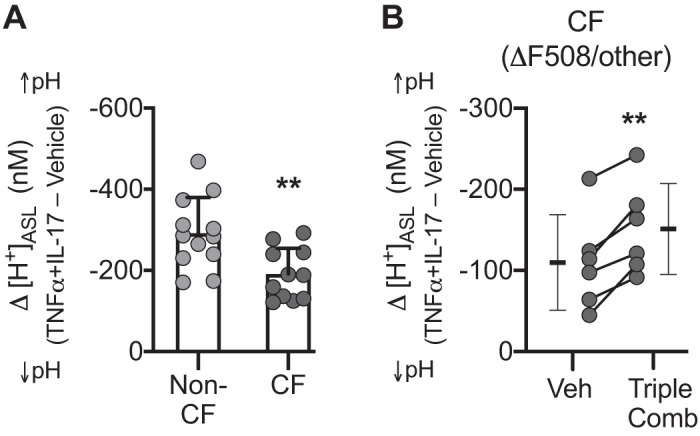
Non-CFTR mechanism(s) mediate TNFα+IL-17-induced airway surface liquid (ASL) alkalinization and operate in tandem with CFTR. Human airway epithelia were treated with TNFα+IL-17 for 24 h, and pH of ASL (pH_ASL_) was measured with SNARF-1-dextran in the presence of ${\text{HCO}}_{3}^{-}$/CO_2_. pH_ASL_ values were converted to [H^+^]_ASL_, and net alkalinization was calculated as the difference (Δ[H^+^]_ASL_) between control and TNFα+IL-17-treated epithelia. *A*: TNFα+IL-17-induced alkalinization in non-cystic fibrosis (CF) vs. CF epithelia (*n* = 12 different donors for non-CF and 11 different donors for CF group). *B*: TNFα+IL-17-induced alkalinization in CF donors carrying at least one *F508-CFTR* allele. Epithelia were treated with either vehicle (DMSO) or the recently approved triple combination of CFTR correctors (3 μM VX-445, 18 μM VX-661, 1 μM VX-770) for 48 h, with the addition of cytokines for the last 24 h (*n* = 6). CF epithelia in *A* and *B* were from different donors and were studied at different times. Bars indicate means ± SD. Groups were compared with unpaired (*A*) or paired (*B*) Student’s *t* test. **P < 0.01.

Because CF epithelia lack functional CFTR, the response to TNFα+IL-17 will depend entirely on non-CFTR ${\text{HCO}}_{3}^{-}$ secretion. To test for a non-CFTR mechanism, we compared TNFα+IL-17-induced responses in non-CF and CF epithelia. We converted pH values (log scale) to [H^+^] (linear scale) using the relation [H^+^] = 10^−pH^ and calculated net alkalinization, i.e., the decrease in D[H^+^]_ASL_ after TNFα+IL-17 treatment. D[H^+^]_ASL_ decreased in both non-CF and CF epithelia (Fig. 4*A*). However, the decrease was larger in non-CF epithelia. This result supported the presence of a CFTR-independent mechanism of alkalinization. It also led us to predict that restoring CFTR function to CF epithelia would further augment the TNFα+IL-17-induced response. To test this possibility, we used epithelia from CF donors carrying at least one F508-*CFTR* allele and treated them with the triple CFTR modulator regimen (VX-445, VX-661, and VX-770) (40). TNFα+IL-17 produced a greater decrease in D[H^+^]_ASL_ in epithelia treated with CFTR modulators (Fig. 4*B*). These results indicate that TNFα+IL-17-induced ASL alkalinization involved CFTR plus one or more non-CFTR ${\text{HCO}}_{3}^{-}$ transporters.

#### TNFα+IL-17 induce pendrin-mediated HCO_3_^−^ secretion.

**Fig. 5.**
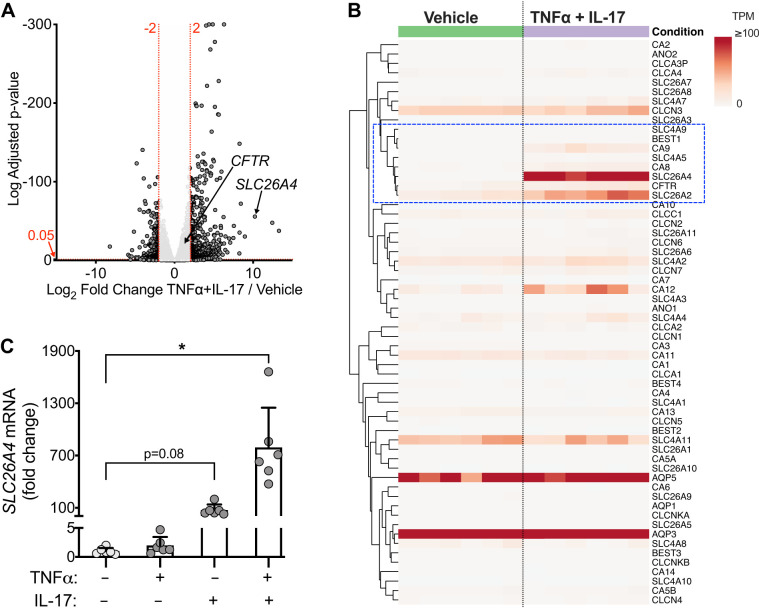
Gene expression profiling identifies *SLC26A4* (pendrin) as a key non-CFTR ${\text{HCO}}_{3}^{-}$ transporter upregulated by TNFα+IL-17. Human airway epithelia were treated with vehicle or TNFα+IL-17 for 48 h, and RNA-sequencing was performed (*n* = 6 different donors). *A*: volcano plot shows TNFα+IL-17-induced gene expression changes. Each data point corresponds to a gene. A horizontal dashed line labeled 0.05 represents arbitrary cutoff of adjusted *P* < 0.05. Two vertical dashed lines labeled −2 and 2 represent arbitrary cutoffs of log_2_ fold change of −2 or less (*left*) and 2 or greater (*right*). Locations of *CFTR* and *SLC26A4* transcripts are shown with arrows. *B*: differential expression of 60 ${\text{HCO}}_{3}^{-}$ transport-related genes displayed as a heatmap of raw transcripts per million reads (TPM). Columns represent epithelia from different donors, and rows represent individual genes. Heatmap and gene clustering were generated using ClustVis tool (see materials and methods). Dashed rectangle highlights a subgroup of ${\text{HCO}}_{3}^{-}$ transport-related genes upregulated by TNFα+IL-17 treatment. *C*: real-time PCR validation of increased *SLC26A4* expression after 24 h of TNFα+IL-17 (*n* = 6 per group). Bars indicate means and SD. *P < 0.05 by repeated measures ANOVA and Dunnett’s multiple comparisons test.

Both TNFα and IL-17 have been reported to modify transcriptional activity in epithelia (17, 47). To identify ${\text{HCO}}_{3}^{-}$ transport mechanisms transcriptionally upregulated by inflammation, we performed RNA-seq. TNFα+IL-17 altered expression of hundreds of genes, as displayed in a volcano plot (Fig. 5*A*). We produced a set of 60 ${\text{HCO}}_{3}^{-}$ transport-related genes using a gene ontology approach and individual gene curation including genes relevant to epithelia (Fig. 5*B*). TNFα+IL-17 increased mRNA for a subset of the genes, including CFTR and members of the SLC26, SLC4, and carbonic anhydrase families. The ${\text{HCO}}_{3}^{-}$ transport-related gene that showed the largest increase in expression was *SLC26A4* (Fig. 5, *A* and *B*). To validate the RNA-seq results, we measured *SLC26A4* expression with quantitative real-time (qRT)-PCR (Fig. 5*C*). TNFα alone did not induce *SLC26A4* expression, whereas IL-17 alone increased it by 74-fold. Remarkably, TNFα+IL-17 increased *SLC26A4* expression by 790-fold, suggesting synergy between TNFα and IL-17 pathways at the level of gene expression.

**Fig. 6.**
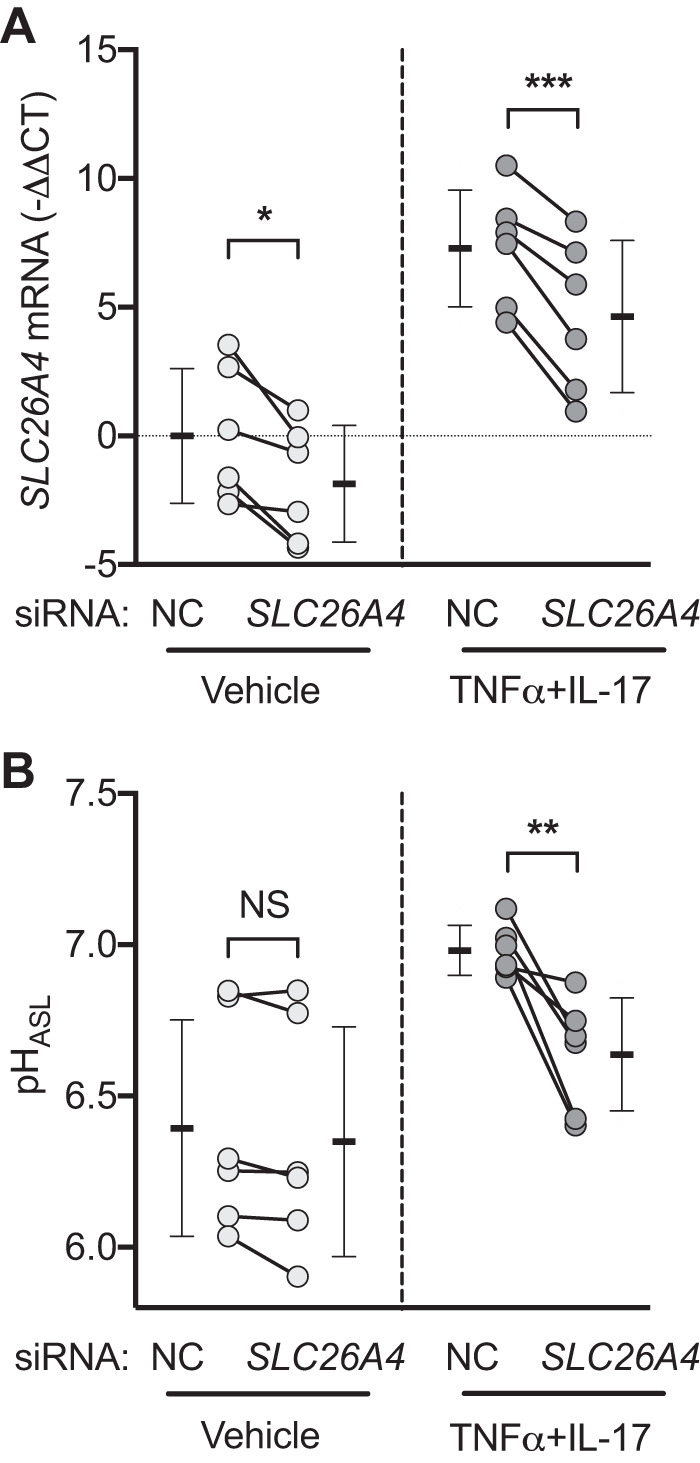
*SLC26A4* (pendrin) contributes to TNFα+IL-17-induced airway surface liquid (ASL) alkalinization. *A* and *B*: siRNA directed against *SLC26A4* was used to knock down expression, and pH of ASL (pH_ASL_) was measured after treatment with either vehicle or TNFα+IL-17 for 24 h (*n* = 6). Individual data points in each group represent epithelia from a different donor. Bars indicate means ± SD. Groups were compared with paired Student’s *t* test. *P < 0.05, **P < 0.01, ***P < 0.001. NC, negative control; NS, not significant.

These results suggested that *SLC26A4* might be a key non-CFTR ${\text{HCO}}_{3}^{-}$ transporter particularly relevant to inflamed airways. *SLC26A4* encodes pendrin, an apical membrane, DIDS-insensitive, electroneutral, Cl^−^/${\text{HCO}}_{3}^{-}$ exchanger (78, 83). To test whether *SLC26A4* (pendrin) was involved in the TNFα+IL-17-induced pH_ASL_ response, we knocked down *SLC26A4* (Fig. 6*A*). *SLC26A4* knockdown did not alter baseline pH_ASL_, but it significantly curtailed the response in TNFα+IL-17-treated epithelia (Fig. 6*B*). This result suggested that pendrin-mediated ${\text{HCO}}_{3}^{-}$ secretion contributed to TNFα+IL-17-induced pH_ASL_ increase. In conjunction with *CFTR* knockdown studies, it also suggested that the maximal pH_ASL_ response to TNFα+IL-17 required CFTR as well as pendrin.

**Fig. 7.**
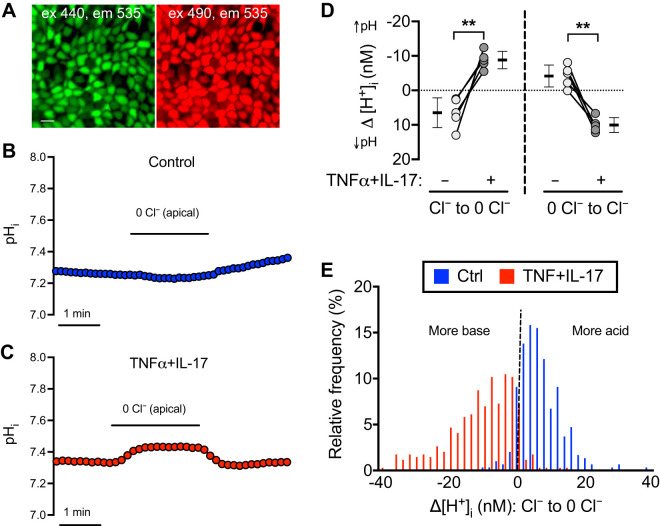
TNFα+IL-17 induce apical membrane Cl^−^/${\text{HCO}}_{3}^{-}$ exchange. Human airway epithelia were treated with either vehicle or TNFα+IL-17 for 48 h. Epithelia were washed, loaded with BCECF, and fluorescence measured with a confocal microscope. *A*: BCECF-loaded airway epithelia viewed en face. Scale bar, 10 μm. *B* and *C*: intracellular pH (pH_i_) responses to varying apical buffer composition in control (blue) and TNFα+IL-17-treated (red) epithelia. *D*: pH_i_ values were converted to [H^+^]_i_ and used to calculate net flux (Δ[H^+^]_i_) in response to removal (*left*) and replenishment (*right*) of Cl^−^ in the apical perfusion buffer (*n* = 5 different donors). Bars indicate means ± SD. Groups were compared with paired Student’s *t* test. **P < 0.01. *E*: Δ[H^+^]_i_ in response to Cl^−^ removal from apical perfusion buffer was calculated at single-cell level by drawing regions of interest around single cells in BCECF-loaded epithelia. Results from 297 cells from control epithelia and 347 cells from TNFα+IL-17-treated epithelia were plotted as frequency distribution. Both distributions were nonnormal by Anderson–Darling test for normality (*P* < 0.0001).

To test for additional functional effects of pendrin expression, we loaded epithelia with BCECF-AM and measured pH_i_ responses using confocal microscopy (Fig. 7*A*). The basolateral solution was Krebs-Ringer with ${\text{HCO}}_{3}^{-}$/CO_2_. We perfused the apical side first with the Krebs-Ringer solution, then substituted Cl^−^ with gluconate until pH_i_ reached a plateau, and then returned to the original Krebs-Ringer solution. If apical Cl^−^/${\text{HCO}}_{3}^{-}$ exchange activity is present, removing apical Cl^−^ should decrease ${\text{HCO}}_{3}^{-}$ exit, increase cytosolic ${\text{HCO}}_{3}^{-}$ concentration, and increase pH_i_. In control epithelia, substituting gluconate for Cl^−^ slightly decreased pH_i_, whereas it increased pH_i_ in TNFα+IL-17-treated epithelia (Fig. 7, *B* and *C*). Changes in acid/base transport are shown in Fig. 7*D* as the net change in H^+^ flux (Δ[H^+^]_i_); removing apical Cl^−^ decreased Δ[H^+^]_i_, i.e., alkalinized cytosol in TNFα+IL-17-treated epithelia. Reintroducing apical Cl^−^ produced the opposite changes (Fig. 7, *B*–*D*). These results suggest that apical Cl^−^/${\text{HCO}}_{3}^{-}$ exchange activity was minimal under basal conditions but was significantly induced by TNFα+IL-17. In conjunction with RNA-seq, qRT-PCR, and knockdown studies, these results identified pendrin as a major non-CFTR ${\text{HCO}}_{3}^{-}$ secretion mechanism induced by TNFα+IL-17.

#### TNFα+IL-17 increase pendrin expression in secretory cells.

We asked whether apical Cl^−^/${\text{HCO}}_{3}^{-}$ exchange activity was uniformly distributed across all cells that reach the apical surface by measuring the Δ[H^+^]_i_ response to apical Cl^−^ removal in individual cells. We recorded responses from 297 control and 347 TNFα+IL-17-treated cells (*n* = 5 different donors per group) and plotted results as a frequency distribution (Fig. 7*E*). TNFα+IL-17-treated cells achieved a greater mean reduction in D[H^+^]_i_. However, the distribution of cellular responses was not normal for either condition (*P* < 0.0001 by Anderson–Darling test for normal distribution). The wide range and skewness of the TNFα+IL-17 distribution indicated heterogeneity of apical Cl^−^/${\text{HCO}}_{3}^{-}$ exchange activity and suggested that a subgroup of cells exhibited a relatively high level of Cl^−^/${\text{HCO}}_{3}^{-}$ exchange. Therefore, we predicted that TNFα+IL-17 might induce pendrin expression in a specific cell type.

**Fig. 8.**
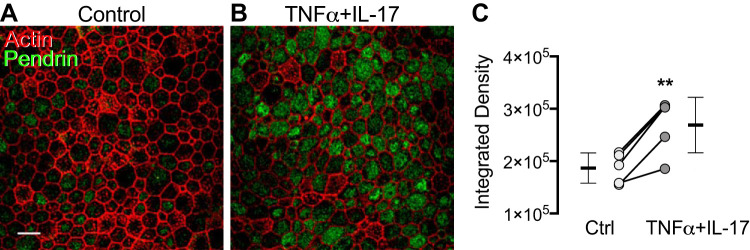
TNFα+IL-17 increase pendrin expression. Human airway epithelia were treated with either vehicle or TNFα+IL-17 for 48 h. *A* and *B*: confocal images show immunostaining for pendrin (green) and actin (phalloidin, red) in control (*A*) and TNFα+IL-17-treated epithelia (*B*). Scale bar, 10 μm. *C*: intensity of pendrin immunolabeling measured as integrated density using the ImageJ software (*n* = 5). **P < 0.01 by paired Student’s *t* test.

**Fig. 9.**
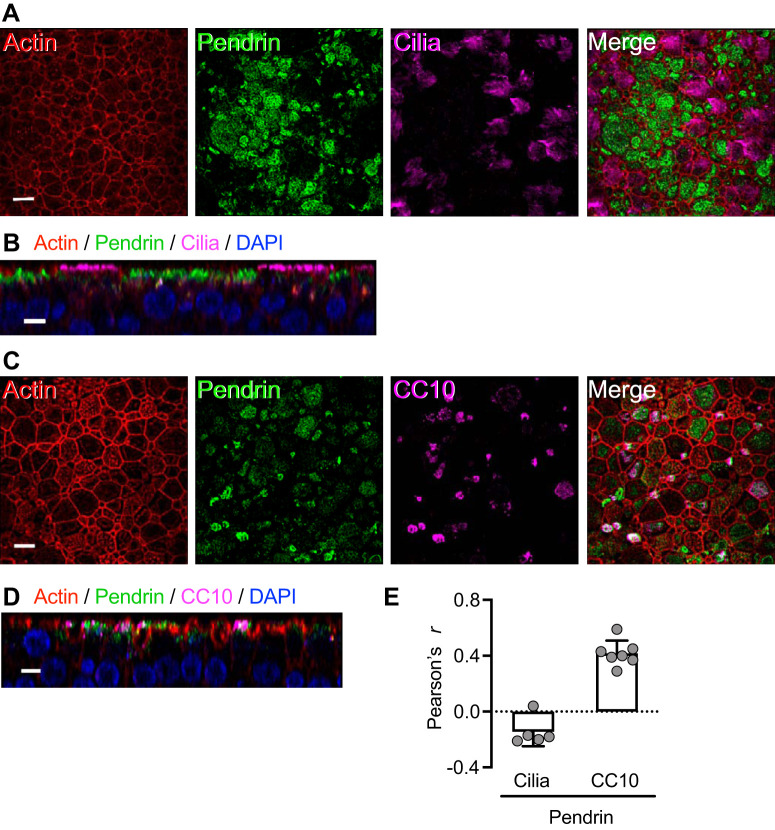
TNFα+IL-17 induce pendrin expression mainly in secretory cells. Human airway epithelia were treated with TNFα+IL-17 for 48 h. *A* and *B*: ciliated cells lacked significant pendrin expression. *A* shows an en face projection, and *B* shows an X-Z projection. *C* and *D*: pendrin-expressing cells were, in many cases, CC10+, which labels secretory cells. *C* shows an en face projection, and *D* shows an X-Z projection. Scale bars: *A* and *C*, 10 μm; *B* and *D*, 5 μm. *E*: colocalization of pendrin with ciliated cell and CC10 markers measured using the ImageJ software and reported as Pearson’s correlation coefficient *r* (*n* = 5–7).

To test this prediction, we immunolocalized pendrin and detected minimal pendrin expression under basal conditions (Fig. 8*A*). However, TNFα+IL-17 markedly increased pendrin immunolabeling (Fig. 8, *B* and *C*), consistent with the transcript data. Interestingly, not all cells expressed pendrin to the same extent. To identify cell types with high-level pendrin expression in TNFα+IL-17-treated epithelia, we colabeled with cell type-specific markers. Ciliated cells (labeled with acetylated-α-tubulin antibody) revealed little pendrin immunostaining (Fig. 9, *A*, *B*, and *E*). In contrast, secretory cells (labeled with CC10 antibody) showed substantial pendrin-immunolabeling (Fig. 9, *C*–*E*).

**Fig. 10.**
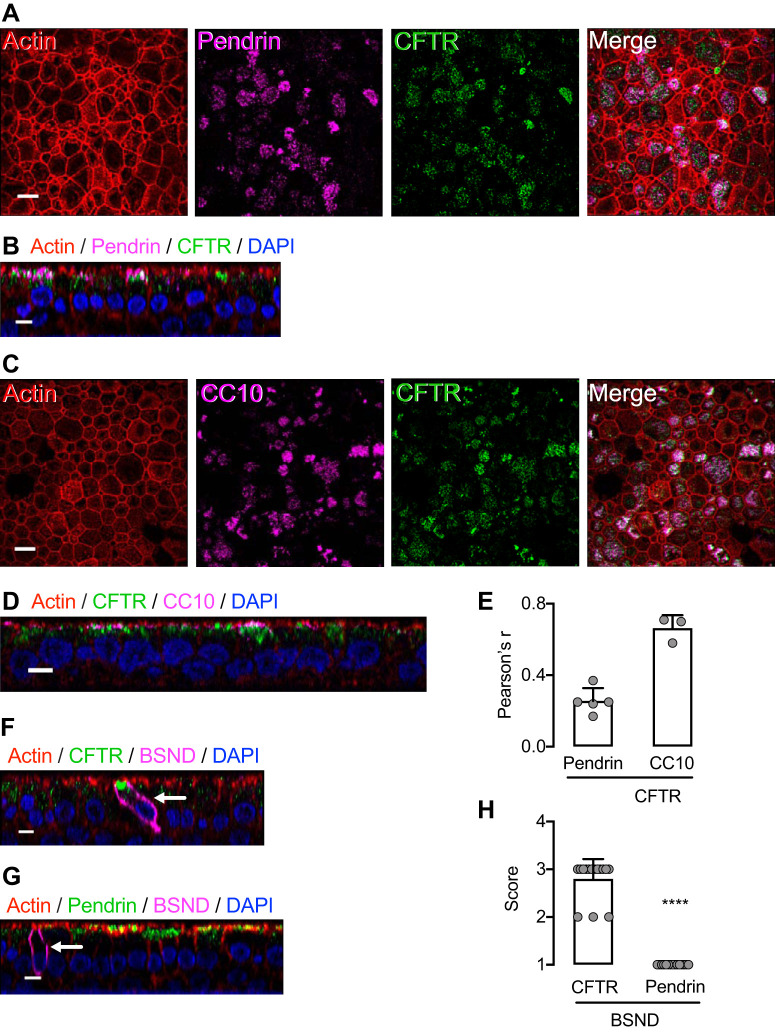
TNFα+IL-17 induces pendrin coexpression with CFTR in secretory cells but not in ionocytes. Human airway epithelia were treated with TNFα+IL-17 for 48 h. *A* and *B*: pendrin was coexpressed with CFTR. *A* shows an en face projection, and *B* shows an X-Z projection. *C* and *D*: CFTR-expressing cells were CC10+ secretory cells. *C* shows an en face projection, and *D* shows an X-Z projection. *E*: colocalization of CFTR with pendrin and CC10 markers measured using the ImageJ software and reported as Pearson’s correlation coefficient *r* (*n* = 3–5). *F* and *G*: ionocytes were identified by labeling with BSND (Barttin) antibodies. Ionocytes showed CFTR labeling but lacked significant pendrin labeling. Scale bars: *A* and *C*, 10 μm; *B*, *D*, *E*, and *F*, 5 μm. *H*: quantification of CFTR or pendrin expression in BSND+ cells. BSND+ cells from CFTR- or pendrin-labeled epithelia were shown in a blinded fashion to 3 investigators experienced in confocal microscopy of airway epithelia. Investigators graded intensity of apical protein (CFTR or pendrin) expression on a scale: none or low = 1, moderate = 2, high = 3. Bars indicate means ± SD. ****P < 0.0001 by Mann–Whitney *U* test.

SLC26 transporters in epithelia may function in concert with CFTR to mediate ${\text{HCO}}_{3}^{-}$ secretion (41, 42). Previous reports suggest that interactions may be structural, functional, or both. We therefore asked whether CFTR and pendrin are expressed in the same cells of TNFα+IL-17-treated epithelia. We found that like pendrin, CFTR was expressed in CC10-positive cells and that it colocalized with pendrin (Fig. 10, *A*–*E*). Recent reports have shown that CFTR is expressed at a very high level in a rare cell type called the pulmonary ionocyte (62, 68). We labeled epithelia with Barttin (BSND), a pulmonary ionocyte marker (68), and confirmed intense CFTR immunostaining at the apical pole (Fig. 10, *F* and *H*). However, we did not detect high-level pendrin expression in ionocytes (Fig. 10, *G* and *H*). Thus, of cell types that reach the apical surface, the cells that express both pendrin and CFTR were predominantly secretory cells.

## DISCUSSION

Our results indicate that TNFα and IL-17, two key regulators of neutrophilic inflammation, markedly increase pH_ASL_ by increasing transepithelial ${\text{HCO}}_{3}^{-}$ secretion. These proinflammatory cytokines increased the expression of two apical ${\text{HCO}}_{3}^{-}$ transporters, CFTR anion channels and pendrin Cl^−^/${\text{HCO}}_{3}^{-}$ exchangers. Moreover, the maximal increase in pH_ASL_ required the activity of both.

At the tissue level, TNFα+IL-17 uniformly increased ${\text{HCO}}_{3}^{-}$ secretion across epithelia from different donors, whereas at the cellular level there was significant cell-to-cell variability in apical Cl^−^/${\text{HCO}}_{3}^{-}$ exchange activity. This heterogeneity was matched by our immunolocalization results, which showed that TNFα+IL-17 increased pendrin expression predominantly in secretory cells and not ciliated cells. Immunostaining of TNFα+IL-17-treated epithelia also revealed that secretory cells coexpressed pendrin and CFTR. These data are consistent with single-cell RNA sequence data from human airway epithelia that showed CFTR and pendrin expression in secretory cells under basal conditions (68, 90). Coexpression of two apical ${\text{HCO}}_{3}^{-}$ transporters suggests that secretory cells are a main cell type contributing ${\text{HCO}}_{3}^{-}$ secretion under inflamed conditions. It will be important for future studies to characterize the cytoplasmic and basolateral membrane mechanisms that supply ${\text{HCO}}_{3}^{-}$ for apical transporters. In this regard, our bulk RNA-seq data showed that TNFα+IL-17 increased members of SLC4 and carbonic anhydrase families. A particularly interesting example is *CA12*. Loss of function of carbonic anhydrase XII has been linked to a CF-like pulmonary phenotype (46).

Ionocytes are another cell type that, although relatively rare, express very high levels of *CFTR* mRNA (62, 68). Our finding of intense CFTR immunostaining in ionocytes confirmed those data. However, we did not detect significant pendrin immunostaining in ionocytes either at baseline or after TNFα+IL-17 treatment. Thus, whether ionocytes secrete substantial amounts of ${\text{HCO}}_{3}^{-}$ and contribute to pH_ASL_ regulation in inflamed airways remains uncertain and may await studies using live-cell markers of ionocytes.

Colocalization of pendrin and CFTR at the apical membrane of secretory cells could enable interactions between the two. Previous data indicate that pendrin can interact with CFTR, and the interaction may enhance activity of both transporters. The basis for such an interaction may be a structural association between the R domain of CFTR and the sulfate transporter and anti-σ factor antagonist (STAS) domain of pendrin (28, 31, 41, 42, 78). Additional studies are needed to fully understand the underlying basis of CFTR-pendrin interactions; increasing pendrin and CFTR expression with TNFα+IL-17 could provide a useful model.

Previous studies found that IL-17 increased pendrin expression and ${\text{HCO}}_{3}^{-}$ secretion in human airway epithelia, and IL-17 was reported to increase pH_ASL_ in epithelia from three donors (3, 44). These data are consistent with our results, with the exception that although we found that TNFα+IL-17 markedly increased pH_ASL_, IL-17 alone did not. This difference is likely due to experimental differences between the studies. Other studies have shown increased pendrin expression after treatment with type 2 cytokines such as IL-4 or IL-13 (30, 33, 48, 76). However, pH_ASL_ responses across studies have not always been consistent. For example, while Gorrieri et al. (30) and Lennox et al. (48) reported that IL-4 or IL-13 increased pH_ASL_, Haggie et al. (33) reported the opposite effect of IL-13, i.e., pH_ASL_ acidification after IL-13 treatment. Differences between cytokines and doses may be responsible, at least in part. Note, for example, that we found TNFα alone induced H^+^ secretion, but when paired with IL-17 it synergistically increased ${\text{HCO}}_{3}^{-}$ secretion. The relative balance between H^+^ and ${\text{HCO}}_{3}^{-}$ secretion under differing culture and experimental conditions may also explain discordant results.

Our study has limitations. First, we used primary cultures of differentiated human airway epithelia. Although this model has yielded numerous insights into airway epithelial biology and enabled the discovery and clinical translation of therapeutics, it does not replace the need for in vivo studies in humans. Second, we studied the effect of two cytokines, TNFα and IL-17, relevant to neutrophil-predominant airway inflammation. Others have reported responses to type 2 cytokines (30, 33, 41, 48). Although previous work has shown interactions between cytokine pathways, including TNFα and IL-17 (14, 57, 74), and multiple cytokines mediate inflammatory response in vivo, to our knowledge the effect of more than one cytokine has not been explored on electrolyte transport or pH_ASL_. However, none of these studies or our data capture the full complexity of the inflammatory environment that may obtain in vivo. Third, we mimicked inflammation using cytokines; it will be important to know the in vivo contribution of inflammatory cells (macrophages, neutrophils, etc.) and/or infectious agents to pH_ASL_ regulation. Fourth, in addition to our study of mRNA, siRNA knockdown, pharmacological inhibition, function, and immunolocalization, quantifying amounts of pendrin and CFTR protein would be of value. Fifth, we generated epithelia from tracheae and proximal large bronchi. However, characterization of epithelial responses in distal small airways will also be of interest.

Inflammation is critical for host defense. Previous studies indicate that in the respiratory system, an acidic pH impairs, and an alkaline pH enhances two host defenses against bacteria, mucociliary transport and antimicrobial activity (1, 2, 9, 19, 66, 77, 80, 88). Finding that TNFα and IL-17, important cytokine regulators of neutrophilic inflammation, increase pH_ASL_ emphasizes the importance of an increased pH_ASL_ in defending the respiratory tract. In this regard, it is interesting to note that humans with inherited, autosomal recessive mutations in pendrin, i.e., Pendred syndrome, have not been reported to have increased risk of adverse respiratory outcomes (54, 82). It may be that CFTR alone is sufficient to support airway host defense. Our studies revealed little pendrin expression under basal conditions, with the result that pendrin knockdown did not alter pH_ASL_. However, TNFα+IL-17 markedly increased pendrin transcripts, functional activity, and immunostaining, and pendrin knockdown reduced pH_ASL_. Thus, maximal pH_ASL_ alkalinization under inflamed conditions may require pendrin as well as CFTR. Systematic large data analyses might define whether people with Pendred syndrome have an increased risk of respiratory outcomes.

The cytokine-induced increased expression of both pendrin and CFTR in serous cells and the resulting increased pH_ASL_ may have implications for therapeutic approaches. For gene therapy/editing approaches in CF, increasing CFTR expression in serous cells or cells that could differentiate to serous cells might be beneficial for raising pH_ASL_. For CF and other diseases with bacterial infection or impaired mucociliary transport, increasing pendrin expression might at least partially increase pH_ASL_ and enhance respiratory host defenses (49).

## GRANTS

This work was supported by the National Institutes of Health (National Heart, Lung, and Blood Institute Grants HL051670 and HL091842), a Cystic Fibrosis Foundation Research Development Program, a Cystic Fibrosis Foundation RDP pilot award to T. Rehman, and a National Institutes of Health training award (HL007638) to I. M. Thornell. I. M. Thornell is supported by the Gilead Sciences Research Program in Cystic Fibrosis, and M. J. Welsh is an investigator of the Howard Hughes Medical Institute.

## DISCLOSURES

No conflicts of interest, financial or otherwise, are declared by the authors.

## AUTHOR CONTRIBUTIONS

T.R. and M.J.W. conceived and designed research; T.R., G.S.R.I., P.H.K., and P.T. performed experiments; T.R., I.M.T., A.A.P., A.L.T., G.S.R.I., P.H.K., P.T., and M.J.W. analyzed data; T.R., I.M.T., G.S.R.I., P.H.K., P.T., M.E.D., and M.J.W. interpreted results of experiments; T.R. prepared figures; T.R. and M.J.W. drafted manuscript; T.R., I.M.T., A.A.P., A.L.T., G.S.R.I., P.H.K., P.T., M.E.D., and M.J.W. edited and revised manuscript; T.R., I.M.T., A.A.P., A.L.T., G.S.R.I., P.H.K., P.T., M.E.D., and M.J.W. approved final version of manuscript.
